# Characteristics of Patients With Adult-Onset Dermatomyositis at 2 Tertiary Care Centres in Ontario, Canada

**DOI:** 10.1177/12034754241301409

**Published:** 2024-11-26

**Authors:** Dea Metko, Dimitra E. Bednar, Fares Alkhayal, Viktoria Pavlova, Kimberly Legault, Mohannad Abu-Hilal

**Affiliations:** 1 Michael G. DeGroote School of Medicine, Hamilton, ON, Canada; 2 Division of Dermatology, University of Toronto, Toronto, ON, Canada; 3 Division of Dermatology, McMaster University, Hamilton, ON, Canada; 4 Division of Rheumatology, McMaster University, Hamilton, ON, Canada

**Keywords:** adult, cutaneous, dermatomyositis, lung, myopathy

## Abstract

Dermatomyositis (DM) is an idiopathic inflammatory myopathy characterized by progressive muscle weakness and distinctive cutaneous findings. The exact incidence and prevalence of DM in the general population is largely unknown, and data on demographic and clinical features in patients in Canada are lacking. This study aimed to comprehensively evaluate the patients with DM in Southwestern Ontario, Canada. A retrospective chart review was conducted for patients with adult-onset DM at 2 tertiary care centres in Ontario, Canada, over a 13 year period. One hundred fourteen patients were included. The mean age was 59, and 80% of patients were female. Skin and muscle involvement coincided in 63% of patients, while skin predated muscle involvement in 11%. Most common characteristic skin manifestations included Gottron’s papules (69%), Heliotrope sign (66%), V sign (61%), and Shawl sign (56%). Among the 114 patients, 88 (77%) were myopathic while 26/114 (23%) were clinically amyopathic dermatomyositis. In the myopathic type, upper extremity weakness, lower extremity weakness, and dysphagia were present in 87%, 81%, and 29% of patients, respectively. Elevated creatine kinase, C-reactive protein, lactate dehydrogenase, and erythrocyte sedimentation rate were found in 66%, 35%, 33%, and 32%, respectively. Lung disease was reported in 39%, particularly interstitial lung disease. Other associated features included malignancy, cardiovascular disease, and less commonly gastrointestinal disease. DM is a multifaceted autoimmune disease with distinct cutaneous and muscular findings. Our study results align with the growing body of evidence supporting DM as a complex systemic disease with the potential involvement of other organs such as the pulmonary, cardiovascular, and gastrointestinal systems.

## Introduction

Dermatomyositis (DM) is a rare idiopathic inflammatory myopathy with characteristic cutaneous findings. While the exact incidence or prevalence of DM is not well known, it is widely recognized as a condition predominantly affecting adult women between the ages of 40 and 65.^1,2^ Most affected individuals frequently display variable cutaneous manifestations, which can differ markedly among patients. Additionally, there exists a subset of patients who may present without evident muscle involvement, while others endure severe and incapacitating muscle weakness. Thus, DM encompasses a wide array of clinical phenotypes, underscoring the complexity and diversity of this condition. Additionally, DM frequently co-occurs with various comorbidities that can have a detrimental effect on its prognosis, for example, involving vital organs such as the pulmonary, cardiovascular, and gastrointestinal systems.^3^ Unlike the juvenile-onset counterpart, adult-onset DM (AoDM) is frequently associated with an underlying malignancy, further complicating the clinical course for affected patients.^4^ Our study aimed to comprehensively describe demographics, cutaneous and musculoskeletal manifestations of DM, comorbidities, laboratory/serologic findings including myositis-associated and myositis-specific antibodies, and other diagnostic modalities for adult patients with DM in Southwestern Ontario, Canada. This is one of the largest cohorts used to describe and evaluate adult patients with AoDM in Canada.

## Methods and Materials

### Data Collection

This retrospective study was approved by the Hamilton Integrated Research Ethics Board. Patient records from dermatology and/or rheumatology clinics affiliated with Hamilton Health Sciences, St Joseph’s Healthcare Hamilton, and/or McMaster University at Hamilton, Ontario, were searched from January 2010 until September 2023. Adult patients (≥18 years) with confirmed diagnosis of AoDM were included. Data collected included demographics, cutaneous and musculoskeletal manifestations, comorbidities, and laboratory values including C-reactive protein (CRP), erythrocyte sedimentation rate (ESR), alanine aminotransferase (ALT), aspartate aminotransferase (AST), creatine kinase (CK), and lactate dehydrogenase (LDH). Additionally, we collected serologies including myositis-associated and myositis-specific antibodies, and findings from other diagnostic modalities including skin biopsies, muscle biopsies, electromyography (EMG), and magnetic resonance imaging (MRI).

### Statistical Analysis

Continuous variables were reported as median with range and mean ± standard deviation. Categorical variables were summarized as frequencies and percentages of the study population and by subgroups, where appropriate.

## Results

Table 1 summarizes the demographic and clinical data collected. A total of 114 adult patients with DM were included in the study. Ninety-one (79.82%) were female. Mean age and other demographics are summarized in Table 1. Among the 114 patients, 88 (77%) were myopathic while 26 (23%) were clinically amyopathic dermatomyositis (CADM). A total of 24/26 (92.3%) were clinically amyopathic while 2/26 (7.69%) were hypomyopathic with no clinical evidence of muscle weakness but where other diagnostic modalities demonstrated muscle involvement. Seventy-two patients (63.16%) had muscle and skin symptom onset simultaneously, while 12 patients (10.53%) had skin involvement predating muscle onset. When the dermatologic manifestations predate musculoskeletal manifestations, the average time between the skin and subsequent muscle onset was 1.33 years (range 1-3). Gottron’s papules were the most common skin finding detected in 79 (69.30%) patients. Cutaneous and myopathic manifestations are summarized in Table 1, and Table 2 summarizes information about special diagnostic and serologic tests including myositis-specific and myositis-associated antibodies.

**Table 1.** Demographics and Clinical Manifestations.

Demographic	n, (%)
Mean current age	59.20 years (±17.62)
Female	n = 91, 79.82%
Mean age at diagnosis	50.43 years (±16.76)
Mean age at diagnosis (female)	50.86 years (±17.98)
Mean age at diagnosis (male)	50.14 years (±15.29)
CADM	n = 26/114, 23%
Amyopathic DM	n = 24/114, 21%
Hypomyopathic DM	n = 2/114, 1.8%
Myopathic	n = 88/114, 77%
Simultaneous skin and muscle onset	n = 72, 63.16%
Skin involvement predated muscle onset	n = 12, 10.53%
Skin manifestations	n, %
Gottron’s papules and/or sign	n = 79, 69.30%
Heliotrope sign	n = 75, 65.79%
V sign	n = 69, 60.53%
Shawl sign	n = 64, 56.14%
Psoriatic like changes	
• Psoriasis-like changes on the scalp only	n = 22, 19.30%
• Psoriasis-like changes on extensor surfaces only	n = 44, 38.60%
• Psoriasis-like changes on both scalp and/or extensor surfaces	n = 49, 42.98%
Holster sign	n = 23, 20.18%
Calcinosis cutis	n = 6, 5.26%
Raynaud’s	n = 27, 23.68%
Dilated nail capillary changes	n = 53, 46.49%
Mechanic hands	n = 15, 13.16%
Myopathic manifestations	n = 88
Upper extremities weakness	n = 78/88 (88.6%)
Lower extremities weakness	n = 73/88 (83.0%)
Dysphagia	n = 26/88 (29.5%)

Abbreviations: CADM, clinically amyopathic dermatomyositis; DM. dermatomyositis.

**Table 2.** Special Diagnostic Tests, Laboratory Values and Serologic Testing Including Myositis-Specific and Myositis-Associated Antibodies.

Special diagnostic tests	n, %
Skin biopsy	n = 69, 61
Positive skin biopsy	n = 48/69, 70
Muscle biopsy	n = 61, 54
Positive muscle biopsy	n = 46/61, 75
EMG	n = 67, 58.77
EMG showing classic myopathic changes	n = 55/67, 82.01
MRI	n = 52, 45.61
MRI showing positive myopathic changes	n = 39/52, 75
Chemistry	n, %
Elevated creatinine kinase	n = 75, 65.79
Elevated CRP	n = 40, 35.09
Elevated AST	n = 36, 31.58
Elevated ALT	n = 39, 34.21
Elevated LDH	n = 38, 33.33
Elevated ESR	n = 36, 31.58
Myositis-specific antibody	n, %
Anti-MI2	n = 4, 3.51
Anti-MDA5	n = 11, 9.65
Anti-TIF1 gamma	n = 8, 7.02
Anti-SAE	n = 4, 3.51
Anti-NXP	n = 5, 4.39
Anti-aminoacyl tRNA synthetase (anti-ARS)	n = 14, 12
Anti-Jo1	n = 13, 11.40
Ani-PL7	n = 1, 0.88
Myositis-associated antibody	n, %
ANA	n = 51, 44.74
Scl-70	n = 4, 3.51
Anti-U1-RNP	n = 4, 3.51
Anti-Ro 52/SSA	n = 30, 26.32
Anti-La/SSB	n = 4, 3.51
SMRNP	n = 2, 1.75
RNP	n = 5, 4.39
Anti-Ku	n = 1, 0.88
Pancreatic disease	n = 1, 0.88
GI vasculitis	n = 1, 0.88
Barrett’s esophagus	n = 1, 0.88
Other (major bleeding event)	n = 1, 0.88

Abbreviations: ALT, alanine aminotransferase; ANA, antinuclear antibody; ARS, Aminoacyl-tRNA Synthetase; AST, aspartate aminotransferase; CRP, C-reactive protein; EMG, electromyography; ESR, erythrocyte sedimentation rate; GI, gastrointestinal; LDH, lactate dehydrogenase; MDA5, Melanoma Differentiation-Associated protein 5; MRI, magnetic resonance imaging; NXP, Nuclear Matrix Protein; SAE, Small Ubiquitin-like Modifier; SSA, Sjögren’s Syndrome Antigen A; SSB, Sjögren’s Syndrome Antigen B; RNP, ribonucleoprotein; TIF1, Transcriptional Intermediary Factor 1; tRNA, Transfer RNA.

Aside from myopathy, extracutaneous manifestations and/or comorbidities in our cohort include respiratory (39.47%), cardiovascular (35.96%), and gastrointestinal (GI) (23.68%). Malignancies were found in 38.60%. Table S1 summarizes types of respiratory, cardiovascular, and GI involvement. Interstitial lung disease (ILD) was the most common respiratory disease. Cardiovascular disease was found in almost one-third of patients with hypertension (HTN) being the most common. Other than dysphagia, the most common GI conditions were gastroesophageal reflux disease (GERD), inflammatory bowel disease (IBD), and irritable bowel syndrome (IBS) in 12.3%, 4.39%, and 3.51%, respectively, from the entire cohort.

## Discussion

Here, we present a large retrospective study providing a comprehensive description of demographics, clinical manifestations, laboratory findings, and serologies including myositis-associated and myositis-specific antibodies for a cohort of 114 patients with AoDM in Canada.

Existing literature has highlighted prevalent demographic patterns in individuals afflicted with DM. Retrospective studies have consistently demonstrated a higher incidence of the condition among female patients than their male counterparts.^5^ In one large European study, 90% of patients with DM were female, similar to 80% in our cohort.^6^ The mean age at diagnosis in previous literature has been reported to be between 40 and 60 years old, which aligns with our patients’ calculated mean age of 59.^7,8^

In our study, CADM is observed in 23% of patients. This is consistent with a large population-based study conducted in the United States where CADM comprised approximately 20% of all patients with DM. A comparable study conducted in Minnesota, United States, revealed that 21% of patients with DM were diagnosed with the clinically amyopathic type.^9^

In myopathic DM, the cutaneous manifestations often precede the onset of myopathic symptoms. Classically, myopathic changes often occur within a 6-month period of the first cutaneous manifestations.^10^ However, in our study, simultaneous onset of skin and myopathic symptoms was more prevalent at 63.16%, while skin involvement preceded myopathy in only 10.53% of cases. Myopathic changes occurring more than 6 months after cutaneous manifestations have been coined as “clinically amyopathic evolving into classic myopathic DM” in previous literature. In one previous retrospective study, this progression was found to occur in approximately 10.3% of their study’s cohort, a figure very similar to our findings of 10.53%.^9^

While patients exhibiting characteristic cutaneous and myopathic features may not necessitate further specialized testing for a definitive clinical diagnosis, confirmatory investigations encompass EMG, MRI, and skin and muscle biopsies for histologic examination. In our cohort, 82% of patients exhibited classic myopathic alterations on EMG, and 75% showed positive myopathic changes in MRI. Prior retrospective studies have indicated similar benchmarks for diagnosing DM with EMG (47.6%-95%), and MRI (50%-87%).^11^ Positive skin biopsy and positive muscle biopsy findings were present in 70% and 75% of our patients, respectively. Muscle biopsies showed results consistent with DM in 96% of patients in a Brazilian study.^12^ Other studies have similarly reported a higher rate of positive skin and muscle biopsy findings, with figures of 90% and 92%, respectively.^9^ It is important to note that although the muscle biopsy positivity rates exceed those observed in our study, they often specifically relate to myopathic DM. In contrast, figures from our study were reported for both CADM and myopathic DM, which may explain the variance. The lower rates of skin biopsy positivity in our study may be attributed to the fact that many biopsies were conducted by non-dermatologists, including medical students, residents, rheumatologists, internists, hospitalists, and family physicians. While rheumatologists are knowledgeable about the cutaneous manifestations of DM, others might not be always familiar with these manifestations and therefore may not choose the most appropriate site or may lack the skills to properly perform skin biopsies. For instance, in 10 patients, the skin biopsy finding was reported as a small and/or superficial sample. Furthermore, the results of 36 skin biopsies performed by dermatologists showed a 92% positivity rate.

DM has a variety of cutaneous and myopathic manifestations that have been well documented in previous literature.^13,14^ Gottron’s sign (or papules) has been found to be the most common cutaneous manifestation followed by Heliotrope sign and periungual erythema.^2^ Gottron’s sign (or papules) has been found to be present in anywhere between 49% and 72.5% of patients according to previous studies and was found to be present within this range (69.30%) in our study.^15,16^ Heliotrope sign was found in a slightly larger percentage of patients with DM in our study (65.79%) than in previous retrospective studies (43%-65%).^16^ Interestingly, our study revealed a higher occurrence of both the V sign (60.53%) and shawl sign (56.14%) among patients, in contrast to the existing retrospective studies, which reported their presence in 47.6% and 30.1% of patients, respectively.^17^ Psoriatic-like changes were less commonly prevalent in the literature (4-8.3%) compared with a drastically higher percentage of 43% in our cohort.^18^ This discrepancy may be attributed to variations in the definition of psoriatic-like changes across studies. Overall, our study revealed a greater prevalence of Heliotrope sign, Raynaud’s phenomenon, psoriatic-like changes, V sign, and shawl sign than the existing literature. Conversely, we observed less frequent occurrences of calcinosis cutis. Furthermore, the prevalence of Gottron’s papules, mechanical hands, and Holster sign in our study aligns closely with what has been reported in the literature.^19-21^ Of note, the higher prevalence of Raynaud’s phenomenon could be attributed to the colder climate in Canada for a significant portion of the year.

In keeping with current literature, proximal muscle weakness of the upper extremities was the most common myopathic pattern in our study. Various studies reported proximal muscle weakness in approximately 75% to 79% of patients with DM.^22,23^ On the other hand, dysphagia was observed in roughly one-third of our study cohort, which is notably lower than what has been documented in the current literature (58%).^12^ Other retrospective studies have noted that the presence of dysphagia in patients with DM may be associated with higher likelihood of internal malignancy, poorer prognosis, and impaired quality of life.^24^

Existing literature has underscored the utility of specific biomarkers in the accurate diagnosis of DM. Elevations in CK, ALT, AST, and LDH are well-established in some patients with DM. Of interest is CRP and/or ESR, which are often minimally or mildly elevated, even in patients with active myositis.^25^ Markedly elevated CRP and/or ESR in patients with DM should raise the suspicion of a coexisting infection, underlying malignancy, or severe ILD.^1^

Over the past few years, myositis-specific and myositis-associated antibodies have proved to be excellent diagnostic and prognostic markers for DM, correlating with specific phenotypes.^26^ Anti-aminoacyl tRNA synthetase (anti-ARS) antibodies, particularly anti-Jo1, were the most common myositis-specific antibody detected in our cohort, followed by anti-MDA5 and TIF1 gamma. In another study of patients with DM, anti-MDA5 (11.5%), anti-Jo1 (10.5%) and other anti-ARS (19.4) were the most prevalent antibodies.^20^ In a retrospective study examining myositis-specific antibodies for the diagnosis of idiopathic inflammatory myopathies including DM, anti-Jo1 (16.9%), TIF1-gamma (15.5%), Mi-2 (9.9%), and MDA-5 (5.6%) were the most prevalent.^27^

Lung involvement is a common feature of DM and a significant cause of morbidity and mortality. In our study, lung disease emerged as the most prevalent comorbidity affecting 39% of patients, a figure notably lower than previous research where as many as 61% of patients exhibited some form of lung disease involvement.^28^ Among these cases, ILD was the most frequently encountered. Prior studies have indicated ILD occurs in at least 30% to 40% of cases of DM and most often in association with anti-Jo-1 or another anti-synthetase antibody.^29^ Notably, ILD often co-occurs with DM and may precede clinical myopathy in 7.2% to 37.5% of cases, frequently constituting the primary cause of hospitalization for patients with DM.^29^ Hence, early evaluation for ILD in newly diagnosed patients with DM and determining whether the patient has rapidly progressive ILD is essential as the prognosis can be improved if treatment is initiated early on.^30^ Additionally, antibodies detected in our study, such as anti-MDA-5, anti-Jo1, and anti-PM-Scl antibodies, are associated with the development of ILD.^29,31^ Previous reviews showed that anti-MDA5 antibodies are present in at least 50% of all DM-associated ILD and more than 80% of DM-associated rapidly progressive ILD causing respiratory failure and death.^32^

Furthermore, previous literature has underscored the connection between DM and various other lung conditions including pulmonary arterial hypertension (PAH), pleural disease, respiratory infections, and respiratory muscle weakness.^33^ Murray et al noted that individuals with DM were at increased risk of mortality from pneumonia compared with the general hospitalized population.^34^ Of note, PAH is present in 8% of patients with anti-ARS autoantibodies and as high as 29% in those with anti-PL7 autoantibodies.^35^ Overall, we observed less frequent occurrences of ILD, asthma, restrictive lung diseases, and chronic obstructive pulmonary disease (COPD) than those of in previous literature.^3,28,36^ Furthermore, the prevalence of PAH and PE in our study aligns closely with what has been reported in literature.^37^

Malignancy was the second most associated condition with DM in our cohort. Existing literature has established that DM is associated with an increased risk of malignancy, with some studies citing an up to 6-fold increase in malignancy in comparison with the general population. ^38^ The risk of developing malignancy is highest within the first 2 years of diagnosis but may remain elevated for up to 5 years.^38^ Age was also seen as another risk factor for the development of malignancy alongside DM, with one study presenting that an independent factor associated with an underlying malignancy in patients with DM was an age at diagnosis of over 52 years.^39^ This has been supported in another retrospective study that found mean patient age to constitute a risk factor for the development of malignancy in patients with DM.^40^ The most commonly reported malignancies across various studies include breast, prostate, ovarian, and lung.^41^ Less common but notable malignancies across the literature included melanoma, hematopoietic, pancreatic, stomach, colorectal cancers, and non-Hodgkin lymphoma.^42,43^ In one study, the predominant type of malignancy among men was lung cancer, whereas among women, it was thyroid, breast, and cervical cancer.^44^ In our study, the most common malignancies in women were breast (10.98%), followed by skin (4.40%) and ovarian (3.3%). In men, lung cancer was the most common (17.39%), followed by rectal and prostate cancer (8.7%). The most common malignancies among women and men in our study are consistent with the findings of previous literature (ie, breast, skin, lung, ovarian, and colorectal cancer). However, likely due to the sample size, our study’s findings were not reflective of the sex differences that exist in malignancy types among patients with DM.

Although patients with DM are often not thoroughly evaluated for possible cardiac involvement, cardiovascular involvement is well described in DM. Previous studies showed that the prevalence of cardiac disorders ranges from 9% to 72% in patients with DM.^33^ Therefore, evaluation for possible cardiovascular diseases is considered essential when managing patients with DM. Previous studies have highlighted that subclinical-associated cardiac conditions and/or comorbidities are frequent, including HTN, conduction abnormalities, and arrhythmias. The occurrence of ECG abnormalities in patients with DM is predominantly attributed to conduction irregularities, with a prevalence ranging from 14% to 62% among patients and that was consistent with our finding 39.02%.^45^ Furthermore, subclinical ECG abnormalities have been identified as a potential risk factor for mortality in patients with DM.^46^ HTN has also been noted in previous literature to manifest at a higher frequency in individuals with inflammatory myopathies, including DM (62%) compared with the general population (9%).^47^ Previous studies have shown that DM is associated with the development of atherosclerosis and increases the risk for myocardial infarction (MI). A large, retrospective, population-based study found a nearly 3- and 4-fold increased risk of MI among 350 and 424 patients with incident DM.^48,49^ Moreover, a 2016 study revealed that one-fifth of DM-related hospitalizations were associated with atherosclerotic cardiovascular changes.^47^ Atherosclerosis is linked to the development and worsening of additional cardiac conditions that were observed in the patients of this study, including coronary artery disease, peripheral vascular disease, heart valve disease, and chronic heart failure (HF)/diastolic cardiomyopathy.^50^ One retrospective study from Denmark found patients with DM to have a higher associated risk of HF and other adverse cardiac outcomes, and furthermore, the authors also indicated that patients with both HF and DM have a poorer prognosis.^49^

GI disorders were present in a subset of patients in our study, accounting for 23.68% of cases, including GERD, IBS, IBD, celiac disease, pancreatic disease, GI vasculitis, and Barrett’s esophagus. While GI involvement is common in patients with DM, little exists in literature about this association compared with studies on lung or cardiac ailments. Nevertheless, while comprehensive prospective studies are still scarce for GI conditions assessed in our study, retrospective studies and case series do exist, providing limited support for their association. Although there is no strong association between DM and IBD, our review showed that 4.39% of patients exhibited this comorbidity. In a retrospective study, 1% of patients with DM were diagnosed with IBD, with half of the cases receiving the IBD diagnosis after the DM diagnosis.^51^ GERD is the most common GI manifestation in existing literature, and that aligns with our study. GERD in DM is believed to be due to esophageal muscle involvement and weakness.^52^ In another Brazilian retrospective study, the most prevalent digestive consequence of DM was gastritis, followed by esophageal abnormalities.^12^

Prior retrospective studies have emphasized that the primary causes of mortality in patients with DM encompass respiratory diseases/failure (28.3%); cardiovascular diseases (17.4%) and malignancy (16.7%).^13,53^ Similarly, 1 study found that the 3 most frequent causes of death for patients with DM were respiratory infection, ILD, or both conditions.^54^ These findings align closely with the outcomes of our study, where most causes of death were malignancy (24%), followed by respiratory disease and/or respiratory failure (17%) and cardiac disease (10%).

The study’s strengths are the multi-centre design, and gathering data from dermatology and rheumatology clinics, contributing to a holistic understanding of DM characteristics. The incorporation of 2 tertiary care centres not only expands the sample size but also ensures a more diverse representation of patients, thereby bolstering the generalizability of the findings. Limitations of the study include the sample size, absence of ethnicity data, and the retrospective nature, which may introduce bias in data collection as reliance on existing medical records can lead to missing or incomplete patient information.

## Conclusion

In conclusion, our study confirmed the complex and systemic nature of DM and comprehensively reviewed demographic and clinical features, as well as laboratory and other diagnostic modalities used to evaluate patients with DM. Our findings reinforce the understanding that DM is not limited solely to skin and muscle; it extends to potential involvement and complications in other vital organs, particularly the pulmonary and cardiovascular systems. This systemic perspective underscores the importance of a holistic, multidisciplinary approach in managing this disease. Our research contributes to the foundation for future studies and improved patient care, ultimately enhancing the quality of life for individuals affected by this challenging disease.

## Supplemental Material

sj-docx-1-cms-10.1177_12034754241301409 – Supplemental material for Characteristics of Patients with Adult-Onset Dermatomyositis at 2 Tertiary Care Centres in Ontario, Canada Supplemental material, sj-docx-1-cms-10.1177_12034754241301409 for Characteristics of Patients with Adult-Onset Dermatomyositis at 2 Tertiary Care Centres in Ontario, Canada by Dea Metko, Dimitra E. Bednar, Fares Alkhayal, Viktoria Pavlova, Kimberly Legault and Mohannad Abu-Hilal in Journal of Cutaneous Medicine and Surgery
