# Supplementary material for: Characteristics of Patients With Adult-Onset Dermatomyositis at 2 Tertiary Care Centres in Ontario, Canada
Source: J Cutan Med Surg. 2024 Nov 26;29(2):124–30. doi: 10.1177/12034754241301409 (PMC11979310; doi:10.1177/12034754241301409)
Supplement: sj-docx-1-cms-10.1177_12034754241301409 – Supplemental material for Characteristics of Patients with Adult-Onset Dermatomyositis at 2 Tertiary Care Centres in Ontario, Canada [file sj-docx-1-cms-10.1177_12034754241301409.docx]

Table S1: Lung, Cardiac, and GI diseases. **CAD:** Coronary Artery Disease **CHF**: Congestive Heart Failure **COPD:** Chronic obstructive pulmonary disorder **DCM:** Dilated Cardiomyopathy **ECG**: Electrocardiogram **GERD:** Gastroesophageal Reflux Disease **GI:** Gastrointestinal **HTN:** Hypertension **IBD:** Inflammatory Bowel Disease **IBS:** Irritable Bowel Syndrome **ILD:** Interstitial lung disease **NYD**: Not Yet Determined.

| **Lung disease** | n=45/114, 39% |
| --- | --- |
| ILD | n=28, 24.56% |
| Pulmonary hypertension (total cohort) | n=6, 5.26% |
| PE | n=2, 1.75% |
| COPD | n=4, 3.51% |
| Restrictive lung disease | n=3, 2.63% |
| Pneumothorax | n=1, 0.88% |
| Cystic fibrosis | n=1, 0.88% |
| Asthma | n=4, 3.51% |
| Other: chronic cough NYD, non-specific pulmonary nodules, other hypoxic respiratory disease NYD | n=3, 2.63% |

| **Cardiac disease** | n=41/114, 36% |
| --- | --- |
| HTN | n=20, 17.54% |
| CHF and/or DCM | n=16, 14.04% |
| Peripheral vascular disease | n=1, 0.88% |
| Right sided heart failure/diastolic dysfunction | n=4, 3.51% |
| CAD | n=2, 1.75% |
| ECG Abnormalities | n=16, 14.04% |
| Heart valve disease | n=1, 0.88% |
| Pericardial effusion | n=1, 0.88% |

| **GI disorder** | n=27/114, 24% |
| --- | --- |
| GERD | n=14, 12.3% |
| IBD | n=5, 4.39% |
| IBS | n=4, 3.51% |
| Celiac disease | n=1, 0.88% |
